# Nanoengineered Shape‐Memory Hemostat

**DOI:** 10.1002/smsc.202400321

**Published:** 2024-12-11

**Authors:** Sarah E. Hargett, Giriraj K. Lokhande, Joseph Duran, Zanir Hirani, Lindy K. Jang, Samantha Foster, Kaivalya A. Deo, Sasha George, Mahjabeen Javed, Taylor H. Ware, Duncan J. Maitland, Akhilesh K. Gaharwar

**Affiliations:** ^1^ Department of Biomedical Engineering College of Engineering Texas A&M University College Station TX 77843 USA; ^2^ Materials Engineering Division Lawrence Livermore National Laboratory Livermore CA 94550 USA; ^3^ Interdisciplinary Program in Genetics Texas A&M University College Station TX 77843 USA; ^4^ Department of Material Science and Engineering College of Engineering Texas A&M University College Station TX 77843 USA; ^5^ Center for Remote Health Technologies and Systems Texas A&M University College Station TX 77843 USA

**Keywords:** expandable biomaterials, hemostats, nanocomposites, porous materials, wound healing

## Abstract

Uncontrolled hemorrhage is the predominant cause of preventable combat deaths. Various biomaterials serve as hemostatic agents due to their procoagulant or absorptive activity. However, these biomaterials often lack expansion capabilities, which severely limits use in noncompressible wounds. This study combines a hemostatic nanocomposite with a shape‐memory polymer foam to design a composite material with both hemostatic and physical expansion properties. This composite is fabricated in two formulations: a foam externally coated in a highly concentrated nanocomposite (“coated composite”) and a foam containing a diluted nanocomposite infused throughout its pores (“infused composite”). Both formulations retain the shape‐memory foam's expansion property. Further, the coated composite shows improved fluid uptake (>2‐fold) versus infused composites or foam. The nanocomposite component dissociates from the foam under degradative conditions, with the foam remaining stable for 30 days. Hemostatic studies illustrate that the coated composite reduces the clotting time by ≈20%. Alternatively, the infused composite improves clotting over a larger distance (up to ≈2× distance from the composite). These results signify a modular hemostatic ability: the coated composite reduces clotting and improves fluid uptake, while the infused composite achieves diffuse clotting and maintains mechanical properties. Thus, these materials pose a strong potential for use in noncompressible wounds.

## Graphical abstract

This study presents a nanoengineered hemostatic material with rapid shape recovery, enhanced fluid uptake, and superior clotting ability. The shape‐memory material, along with its biodegradability and biocompatibility, offers a promising solution for managing uncontrolled hemorrhage.

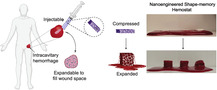

## 1 Introduction

Uncontrolled hemorrhage is the predominant cause of preventable deaths (up to 80%) on the battlefield^[^ ^1^ ^]^ and the second most common cause of civilian trauma deaths.^[^ ^2^ ^]^ Most of these deaths are due to noncompressible, hemorrhagic injuries which typically occur in the torso^[^ ^3^ ^]^ and are not amenable to tourniquet use.^[^ ^4^ ^]^ These injuries bleed profusely, often resulting in death within 30 min.^[^ ^5^ ^]^ Many hemostatic products have been developed to address the challenges of noncompressible wounds, but few have shown effectiveness on the battlefield. These injuries require hemostatic materials with sufficient expansion to fill the wound void and to oppose the blood pressure of a hemorrhaging vessel. XStat (RevMedx, Wilsonville, OR) is a first‐in‐kind hemostatic device cleared by the US Food and Drug Administration for the treatment of noncompressible junctional wounds and consists of 92 compressed cellulose sponges coated with chitosan.^[^ ^6^ ^]^ The sponges are injected into the wound cavity and expand by 12–15‐fold upon contact with blood^[^ ^7^ ^]^ thus providing a physical barrier and exerting pressure on ruptured blood vessels.^[^ ^8^ ^]^ However, XStat use is temporary, with the intended use limited to 4 h, and requires 22‐fold longer surgical removal time than standard gauze.^[^ ^9^ ^]^ These limitations indicate that a similar material with appropriate biodegradation is needed for the treatment of noncompressible injuries.

Engineered biomaterials have been used as hemostatic agents especially when shown to be injectable and highly biocompatible.^[^ ^10^ ^]^ In particular, our previous work with a combination of gelatin and synthetic nanosilicates has demonstrated a 77% reduction in clotting time.^[^ ^11^ ^]^ Gelatin, a denatured form of collagen, has abundant cell adhesive motifs (RGD motifs), absorbs body fluids, and contains positive and negative moieties on its backbone.^[^ ^12^, ^13^ ^]^ Nanosilicate (Na^+^_0.7_[(Mg_5.5_Li_0.3_Si_8_O_20_(OH)_4_)]^−^_0.7_) is a disc‐shaped, synthetic silicate with a dual surface charge.^[^ ^12^, ^13^ ^]^ In addition, nanosilicates have very low cytotoxicity (IC_50 _≈ 4 mg mL^−1^), compared to other nanomaterials such as graphene (IC_50 _≈ 0.1−1 mg mL^−1^), and they dissociate into nontoxic ions (Na^+^, Mg^2+^, Si(OH)_4_, Li^+^) in physiological conditions.^[^ ^11^, ^12^, ^13^, ^14^, ^15^, ^16^ ^]^ Our previous studies showed that this nanocomposite resulted in improved physiological stability and in vitro and in vivo hemostatic performance by enhancing protein adsorption and platelet adhesion.^[^ ^11^, ^13^, ^14^, ^17^ ^]^ Recently, this nanocomposite biomaterial has received FDA approval for use as an embolizing agent in peripheral vasculature.^[^ ^18^ ^]^

Despite these advantages, our previous nanocomposite lacks the ability to expand and cannot contribute the necessary pressure against bleeding vessels. Incorporating a shape‐memory polymer foam can enhance fluid absorption as well as contribute a temperature‐ and fluid‐responsive expansion property. This biomaterial is a promising material for designing hemostatic agents, particularly for noncompressible wounds because of its excellent biocompatibility,^[^ ^19^ ^]^ rapid hemostatic properties,^[^ ^20^ ^]^ and large volume expansion (50–100×).^[^ ^21^, ^22^ ^]^ Shape‐memory foam can be compressed into a compact secondary shape which can be maintained while under its dry glass transition temperature (50–70 °C).^[^ ^23^ ^]^ Once the foam is injected into a wound cavity and contacts blood at body temperature, the foam is plasticized by moisture. As a result, the foam expands and recovers its original porous primary shape. This feature allows the foam to be injected into a deep and narrow wound tract and expand to fill an irregularly shaped cavity. Further, the foam facilitates blood coagulation due to tortuous open cell structure and high surface area‐to‐volume ratio. This shape‐memory foam has received FDA approval for use as an embolic material in peripheral vasculature.^[^ ^24^ ^]^ A combination of compressed and shredded shape‐memory foam has demonstrated considerable success in achieving rapid hemostasis in a porcine model of lethal traumatic hemorrhage.^[^ ^25^ ^]^ This shredded foam demonstrates superior application time, but still is limited in its ability to apply outward pressure.^[^ ^26^ ^]^ This application demonstrates the strong potential of this material to be injected into a wound cavity and rapidly absorb blood.^[^ ^25^ ^]^

In this study, we combine a gelatin‐nanosilicate nanocomposite mixture with a shape‐memory polyurethane foam in two distinct formulations to achieve improved fluid absorption and blood clotting in the resulting composite structure. The presence of nanosilicates also increases the availability of nucleation sites for clot formation, providing enhanced physiological hemostasis in the wound area. Gelatin provides a stable structure to the nanocomposite and provides a microporous architecture within the void spaces of the macroporous shape‐memory foam, thereby enhancing fluid uptake. Herein, we design two composite formulations with varying concentrations of gelatin‐nanosilicate nanocomposite to derive composite structures with appropriate fluid absorption, expansion, and hemostatic ability for noncompressible hemorrhage.

## 2 Results and Discussion

### 2.1 Synthesis and Characterization of Shape‐Memory Hemostats

**Figure 1:**
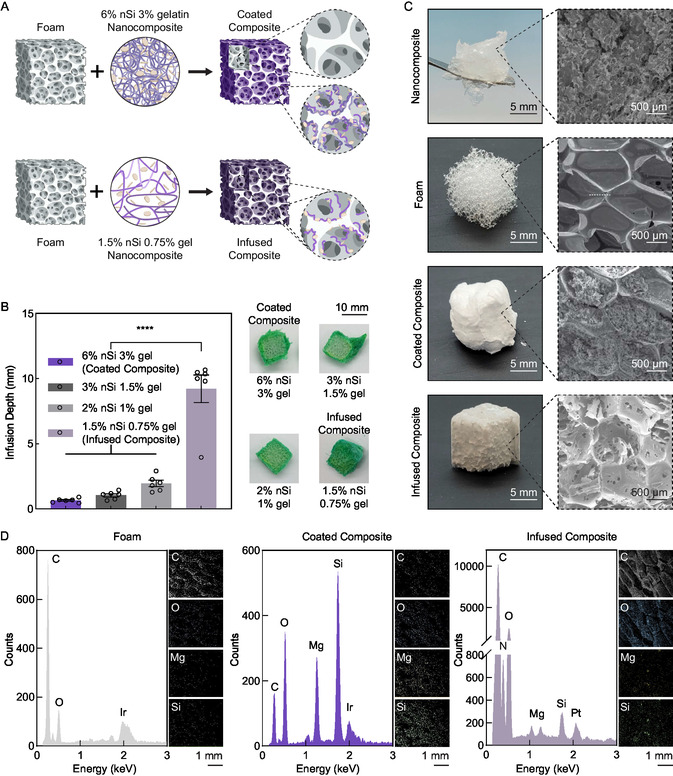
Design and fabrication of a nanoengineered shape memory biomaterial. A) Schematic representation of coated and infused composite fabrication. The figure was produced using Biorender.com. B) Optimization of nanocomposite demonstrating infiltration of nanocomposite within foam's porous structure at various concentrations of nanosilicate (nSi) and gelatin (gel). Representative images are shown. *N* = 6; bars show mean ± standard error of the mean; Ordinary one‐way ANOVA with Tukey multiple comparison test, ****p < 0.0001 (GraphPad Prism 9, exact *p* values are provided in Table S1, Supporting Information). C) Light photographs and SEM images of nanocomposite (6% nSi 3% gelatin), foam, coated composite, and infused composite showing microporous structure of nanocomposite, macroporous structure of foam, and combined macro‐ and microporous structures of coated and infused composites. D) EDS analysis of foam, coated composite, and infused composite highlighting the presence of nanosilicates, indicated by the presence of Mg and Si ions. Representative images and spectra are shown. Abbreviations: nSi ‐ nanosilicate; gel ‐ gelatin.

We first synthesized gelatin‐nanosilicate nanocomposite mixtures as in our previously reported study.^[^ ^11^ ^]^ Briefly, we combined nanosilicates and gelatin in deionized water. This mixture was heated and vigorously stirred in order to produce a homogenous nanocomposite. Separately, a polyurethane‐urea shape‐memory polymer foam with macroscopic porosity was fabricated according to our previously published methods.^[^ ^21^, ^23^ ^]^ In brief, an isocyanate premix composed of hexamethylene diisocyanate (HDI), triethanolamine (TEA), and hydroxypropyl ethylenediamine (HPED) was combined with an alcohol premix composed of TEA and HPED along with blowing agents, catalysts, and surfactants. The ratio of reactants was maintained such that 40% of the isocyanate groups were satisfied by HPED and the remainder by TEA. The resulting foam was then sectioned into cubes, discs, or cylinders. Finally, the polyurethane foam was either externally coated or internally infused with the nanocomposite mixture to produce hemostatic, shape‐memory composites (**Figure** **1**A).

Examination of the nanocomposite's ability to combine with the polyurethane foam revealed that total solid mass concentrations of 3% or more resulted in a nanocomposite that was too viscous to effectively permeate the foam's pores (Figure 1B). Mechanical reticulation of the foam did not appear to have a noticeable impact on the ability of the nanocomposite to permeate the foam. The higher mass concentration resulted in a nanocomposite layer around the foam's outside surface, while lower mass concentrations infiltrated the foam to varying degrees. A diluted nanocomposite (1.5% nanosilicate, 0.75% gelatin) was best able to penetrate the porous polyurethane foam's full thickness. Incubating the composite at 60 °C for 1 h allowed the nanocomposite to fully infuse through the porous foam due to the relaxation of gelatin above its melting temperature.^[^ ^27^ ^]^ The original, highly‐concentrated nanocomposite (6% nanosilicate, 3% gelatin) was used to prepare a “coated composite” with the polyurethane foam while the diluted nanocomposite (1.5% nanosilicate, 0.75% gelatin) was used to prepare an “infused composite” with the polyurethane foam. These composites were held under vacuum and then lyophilized to further integrate the foam and nanocomposite and to enhance the fluid uptake capacity of the composite. Both variations were subsequently evaluated for shape‐memory and other mechanical properties along with hemostatic potential.

Scanning electron microscopy (SEM) imaging (Figure 1C) revealed the successful application of nanocomposite to the porous foam structure. SEM images (Figure 1C) of the foam alone show a macroporous structure with a ≈1000 μm pore diameter. The composite structures display foam pores filled with lyophilized nanocomposite, which presents a much smaller pore diameter (<50 μm) and provides an interconnected, microporous architecture within the foam's void spaces to the extent that the nanocomposite can infiltrate the foam. This increases the surface area of the composites allowing for enhanced fluid retention and contact area. The energy‐dispersive spectroscopy (EDS) spectra (Figure 1D) of the foam showed bands for carbon which decreased in intensity relative to oxygen and other elements in the composites’ spectra. The relatively diminished presence of carbon in the coated composite's EDS spectra can be attributed to the limited penetration depth of EDS.^[^ ^28^ ^]^ This result thus shows that the original nanocomposite has a significant deposited thickness and obscures the foam, while the diluted nanocomposite presents a thinner layer on the foam and within the pores. Additionally, the composites’ spectra show characteristic bands of magnesium (Mg) and silicon (Si) and increased oxygen (O) band intensity (Figure 1D). Both magnesium and silicon are present in nanosilicates but are absent in untreated foams. These results indicate that the composites' fabrication process was successful in incorporating the nanocomposite on and within the foam's porous structure.

### 2.2 Composites Demonstrated Biphasic Mass Loss Due to Dissolution and Degradation

**Figure 2:**
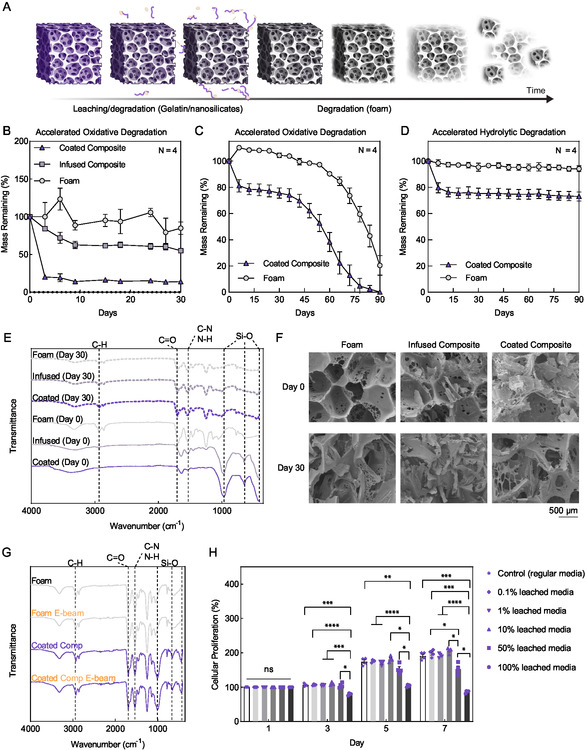
Degradation characteristics and cytocompatibility of foam and composites. A) Schematic of nanocomposite (purple) dissolution and foam (gray) degradation. The figure was produced using Biorender.com. B) Accelerated oxidative degradation of foam, infused composites, and coated composites for up to 30 days. *N* = 4; points show mean ± standard deviation. C) Accelerated oxidative degradation of foam and coated composites up to 90 days. *N* = 4; points show mean ± standard deviation. D) Accelerated hydrolytic degradation of foam and coated composites for up to 90 days. *N* = 4; points show mean ± standard deviation. E) Initial and day 30 FTIR spectra for foam, infused composite, and coated composite with identification of peaks relevant to foam and composite structures. F) SEM images showing porous structure and surface degradation. Representative images are shown. G) FTIR spectra before and after E‐beam sterilization at 43.8 kGy showing maintenance of relevant peaks between non‐ and E‐beam sterilized samples. H) Cellular proliferation following treatment with media containing solutes leached from composite. Cellular proliferation was determined via Alamar Blue assay. *N* = 4; bars show mean ± standard error of the mean (two‐way ANOVA with Tukey post hoc test in GraphPad Prism 9.0. *p < 0.0332, **p < 0.0021, ***p < 0.0002, and ****p < 0.0001). Abbreviations: E‐beam ‐ electron‐beam; comp ‐ composite.

Gravimetric analysis was performed to characterize the degradation profiles of foam, infused composites, and coated composites in both accelerated oxidative conditions and accelerated hydrolytic conditions. In each case, both the infused and coated composites exhibit a sharp initial decrease in mass, followed by a steady maintenance of a lower mass level. This is likely due to rapid dissolution of the nanocomposite component, causing this mass to fall off and leave the foam component behind (**Figure** **2**A) since the physically crosslinked and hydrophilic gelatin and nanosilicates diffuse out easily in 37 °C solution. This result is further supported by the relatively steady mass and lack of degradation of the foam for up to 30 days under both oxidative and hydrolytic conditions (Figure 2B–D). In an extended oxidative study with foam and the coated composites (Figure 2C), the foam showed minimal mass loss until day 54, and then, noticeably faster mass loss was observed. This result is likely due to surface degradation of the foam's membrane and struts prior to day 54 and strut fragmentation which occurs afterward, triggering large mass loss from the bulk of the foam^[^ ^23^ ^]^ (Figure 2C). The polyurethane foam has previously been shown to degrade into lower amines, aldehydes, and carboxylic acids under oxidative conditions due to the cleavage of the C—N bond,^[^ ^23^, ^29^ ^]^ thus contributing to the overall breakdown of the polymeric foam. The hydrolytic condition did not produce notable mass change after the initial dissolution of the nanocomposite from the composite structure (Figure 2D). The mass after this point was steady, indicating that most of the nanocomposite was diffused out of the foam by this point and the foam itself was generally unaffected by the hydrolytic condition. The infused composite was not evaluated in the hydrolytic condition due to observed similarities in the oxidative condition.

Through comparison of the samples’ attenuated total reflectance Fourier‐transform infrared (ATR‐FTIR; FTIR) spectra, peaks at 990, 640, and 430 cm^−1^ were determined to be caused by the presence of the nanocomposite as they were not present in foam spectra (Figure 2E). Composite samples were also analyzed by comparing the spectra throughout the study period (Figure 2E). A steady decrease in transmittance was noted at the 990, 640, and 430 cm^−1^ peaks as the samples progressed through the degradation process. This decrease is attributed to the overall loss of nanocomposite from the composite due to submersion in the fluid bath. The decreasing quantity of nanocomposite remaining in the foams throughout the study lowers the intensity of these peaks, while the relative intensity of the peaks attributed to the foam is seen to increase. This was observed visually in SEM imaging of the samples at days 0 and 30 (Figure 2F) with relatively less nanocomposite present within the foam's pores and visible damage to the foam's structure. Minimal change was noted at peaks specific to the polyurethane foam (i.e., 2900, 1700, and 1540 cm^−1^) between days 0 and 30, supporting the relative stability of the foam component in degradative conditions.

Degradation studies demonstrated that the composite has a similar hydrolytic degradation profile to that of the foam after the initial diffusion of the nanocomposite but has a faster oxidative degradation rate compared to the foam alone. The significant mass drop in the oxidative degradation study, attributed to the breakdown of the foam, was observed 18 days earlier in the coated composite than in the foam. Eighteen days in accelerated oxidative degradation in vitro can be correlated to ≈260 days in vivo in a porcine model according to the calculation from a previous study that compared the in vitro degradation data of similar foams to the in vivo degradation data of implanted foam in a porcine sidewall aneurysm.^[^ ^29^ ^]^ This indicates that the composites can be estimated to degrade within 3.6 years in vivo which is comparable to the degradation rate of previously developed biodegradable foams made with ester‐containing monomers.^[^ ^23^ ^]^ This result is noteworthy in that the foam's degradation rate can be accelerated not only by chemically modifying the foam structure but also by physically coating the foam with a highly hydrophilic nanocomposite to draw water and dissolved reactive oxygen species into the center of the composite structure. For future work, these chemical and physical methods can be combined to attain a hemostatic material that has a faster degradation rate and thus better matches the physiologic wound healing timeframe.

Before assessing the impact on cellular viability, the chemical compositions of foam and the coated composite were analyzed before and after electron beam (E‐beam) sterilization at a radiation dose of 43.8 kGy using ATR‐FTIR (Figure 2G). The FTIR spectra showed that there is no difference following sterilization of neither the foam nor coated composite and suggest that this sterilization method does not alter any chemical structures of the composites. The infused composite was not tested as the foam and coated composite represent the extremes of the designed systems; it can be reasonably inferred that there will not be a change observed in the infused composite formulation, either. This evaluation was repeated for samples which were sterilized by placing them under a 365 nm ultraviolet (UV) lamp to identify an alternative, more accessible method of sterilizing. Again, there was no observed change in the FTIR spectra of the sterilized foam or coated composite, indicating that UV light exposure is a suitable method for sterilizing the composites (Figure S1, Supporting Information).

The sterilized samples were subsequently tested using an indirect exposure assay to determine cytocompatibility (Figure 2H). The original gelatin‐nanosilicate nanocomposite has been extensively studied for its support of the growth of hMSCs in vitro^[^ ^27^, ^28^, ^29^, ^30^ ^]^ and the foam has been shown to be biocompatible in previous studies.^[^ ^11^, ^31^ ^]^ Given the established biocompatible nature of both components, we hypothesized that the composite formulations would result in a cytocompatible scaffold. We evaluated the cytocompatibility of the coated composite as an extreme of the formulations. We incubated composites in media and subsequently treated the cells with varying concentrations of the exposed media. This allowed us to evaluate the impact of leached solutes on cell viability without the accumulation of debris which may have confounded results seen in a transwell assay (Figure S3, Supporting Information). We speculate at lower concentrations, magnesium and silicate ions from the composite surface could be introduced into the medium supplementing hMSC growth, as evidenced by increased cellular metabolic activity and proliferation in days 3–7 when normalized to day 1 (Figure 2H). However, when treated with undiluted exposed media, we observed a minor decrease in cellular proliferation. We speculate that the composite adsorbed media proteins onto the nanosilicates, thus reducing the nutrients present in the exposed media.

### 2.3 Shape‐Memory Hemostats Rapidly Expand under Physiological Conditions

The treatment of noncompressible hemorrhage using injectable hemostatic devices requires both absorbing fluids at the wound site and applying adequate pressure against additional bleeding. Further, bleeding must be controlled rapidly to prevent further blood loss: hemostatic treatment should be delivered within 10 min from the time of injury to prevent the development of hemorrhagic shock^[^ ^30^ ^]^ according to average cardiac output rates for adults.^[^ ^31^ ^]^ The shape‐memory characteristic of the foam allows expansion to return a compressed composite to a larger, primary shape upon contact with fluids at elevated temperatures.^[^ ^23^ ^]^

**Figure 3:**
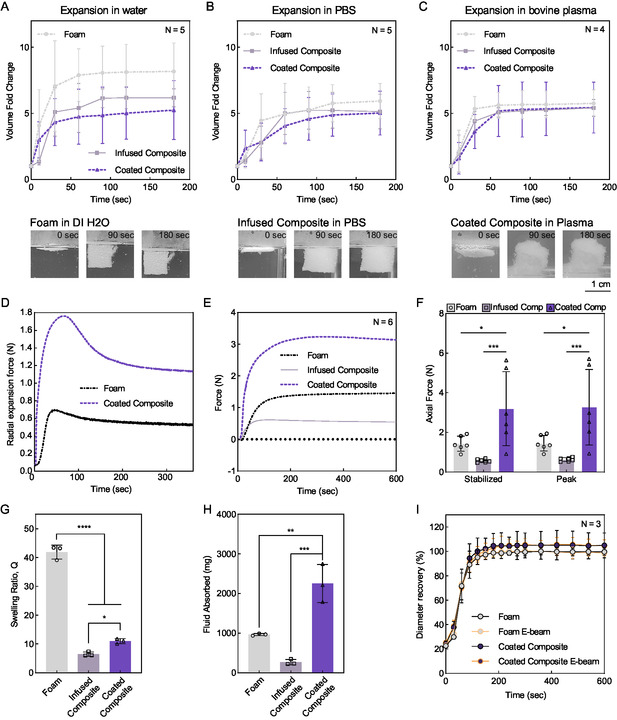
Expansion characterization of composites and foam. A) Expansion of foam, infused composite, and coated composite in deionized water (*n* = 5); points show mean ± standard error of the mean. Representative images of foam are shown. B) Expansion of foam, infused composite, and coated composite in PBS (*n* = 5); points show mean ± standard error of the mean. Representative images of the infused composite are shown. C) Expansion of foam, infused composite, and coated composite in citrated bovine blood plasma (*n* = 4); points show mean ± standard error of the mean. Representative images of the coated composite are shown. D) Radial expansion force when submerged in deionized water (*n* = 5); representative curve shown for visual clarity. E) Axial expansion force when submerged in PBS (*n* = 6); points shown are mean. Standard deviation is not shown for visual clarity. F) Comparison of axial expansion forces. Bar graphs show mean ± standard deviation (two‐way ANOVA with Tukey multiple comparisons test). G) Swelling ratio of foam, infused composite, and coated composite in PBS at 37 °C (*n* = 3); points show mean ± standard deviation (Ordinary one‐way ANOVA with Tukey multiple comparisons test) H) Raw (non‐normalized) fluid absorbed per sample type. Samples are all 1 cm cubes (*n* = 3); points show mean ± standard deviation (Ordinary one‐way ANOVA with Tukey multiple comparisons test). I) Expansion profile of coated composites before and after E‐beam sterilization at 43.8 kGy (*n* = 3); points show mean ± standard deviation. Statistical significance is indicated by *p < 0.0332, **p < 0.0021, ***p < 0.0002, ****p < 0.0001, GraphPad Prism 9.

The shape recovery and expansion of compressed samples demonstrated that the coated composite, infused composite, and foam all expanded to ≈5× their compressed volume once submerged in a liquid at physiological temperature (**Figure** **3**A–C). The expansion of shape‐memory materials upon contact with water has been explored in several novel hemostatic materials.^[^ ^32^ ^]^ The expansion rate was slowed in phosphate buffered saline (PBS) compared to in deionized water (DI H_2_O) and was fastest in bovine plasma; however, the difference in time required for each sample to fully expand was nearly identical, indicating that the shape‐memory property was unaffected by the incorporation of nanocomposite in either the coated or infused formulation and that the composite was relatively insensitive to the presence of solutes and dissolved proteins. Stable expansion was achieved by all samples in all fluids within ≈120 s, which is comparable to other novel hemostatic materials with relatively hydrophobic polymers,^[^ ^32^, ^33^ ^]^ but much slower than others with more hydrophilic polymers.^[^ ^34^ ^]^ In addition, the recovery time for the coated composite in blood plasma was notably faster (<4.5 min for 100% recovery) than that of previously developed polyethylene glycol (PEG)‐based, iodine‐doped hydrogel‐coated foam (>15 min to recover 80% of its shape in 37 °C water).^[^ ^35^ ^]^ This rapid expansion is attributed to the counteracting influences of stronger interaction between the gelatin backbone, which contains polar, hydrophilic groups (–COOH and –NH_2_), and water molecules compared to the interactions between water and polyurethane foam or a PEG‐based hydrogel. Water molecules bound to the hydrophilic groups can open up a transport channel for water to move toward the center of the compressed samples,^[^ ^36^ ^]^ which plasticizes the shape memory polyurethane polymer and accelerates shape recovery. The results indicate that both composite formulations would be appropriate for treating noncompressible wounds within a window of 10–30 min^[^ ^37^ ^]^ and demonstrate improvement over our previous foam‐polymer composites.^[^ ^35^ ^]^

In addition to rapid expansion and shape recovery, the pressure generated through the expansion of the composite must be sufficient to stop bleeding without damaging the surrounding tissues. To this end, we fabricated both cubic and cylindrical samples to evaluate axial and radial expansion forces. Radial expansion force of crimped cylindrical samples (Figure 3D) was measured using the parallel plate method.^[^ ^38^ ^]^ The expansion force increased sharply immediately after the samples contacted water at physiological temperature and reached a peak force within 2 min. This force decreased slightly and then maintained a constant level afterward. The time at which the constant force was reached was consistent with the time point when the crimped samples expanded to their largest diameters in deionized water. This suggests that the fully expanded samples became malleable due to the full plasticization. The average radial maximum force of the coated composite was 1.6 N, which was 2.0× higher than that of the foam. The average force of the coated composite after the force became constant was 1.0 N which was still 1.8× higher than that of the foam. With the assumption that the foam and the composite have the same contact area with the parallel plates, this force corresponds to a pressure of ≈4.8–8.0 kPa, which is at the lower end of gauze pressure range (≈6–26 kPa) measured by Kragh et al. using a wound model made of Perma‐Gel Ballistic Gel.^[^ ^9^ ^]^

Similarly, the axial expansion force demonstrated constant force within 2 min and a similar expansion rate to the radial profile (Figure 3E) for both the infused and coated composites. However, the axial expansion force was notably higher than the radial expansion force. This is likely due to the orientation of the foam's pores,^[^ ^39^ ^]^ with the shorter pore axis in the radial direction and the longer pore axis in the axial direction. The average axial constant force was 3.2 N for the coated composite, which was 2.2× higher than that of the foam (1.4 N), and 0.57 N for the infused composite, which was 40% of the force by the foam but this difference was not statistically significant (Figure 3F). Following the same assumptions as above, the coated composite, infused composite, and foam exerted axial pressures of up to 29.9, 7.6, and 19.4 kPa, respectively. The lower expansion force observed in the infused composite is likely due to the presence of additional material within the foam's structure, which rapidly absorbed water and became viscous, thus restricting the outward movement of the foam. In contrast, the coated composite benefited from the expansion of the dense, hydrogel‐like network on the outer surface of the foam structure to bolster expansion. The axial expansion pressure of the coated composite was noted to be similar to the literature value for gauze pressure and marginally higher than the XStat pressure (16–21 kPa),^[^ ^9^ ^]^ suggesting appropriate expansion forces with minimal risk of tissue damage. The infused composite was not measured in the radial direction due to the decision to proceed with only cube‐shaped particles due to their improved fabrication process. The forces observed for the composites were notably much lower than the expansion forces generated by a polyvinyl acetate/carboxymethylcellulose nanofiber (PVA/CMC) hemostat, which was able to generate forces of at least 4 N,^[^ ^32^ ^]^ but similar to a hemostatic cryogel developed from chitosan and dopamine.^[^ ^40^ ^]^ It would be worth exploring further the tradeoff between high expansion force to plug wounds with the impact of that force on surrounding tissues.

The swelling ratios of the foam, the coated composite, and the infused composite in 37 °C PBS were measured to compare the fluid uptake capacity of the materials themselves (i.e., noncompressed foams and composites) in physiological conditions (Figure 3G). The samples were standardized as 1 cm cubes. The foam alone has a higher swelling ratio than either of the composite structures, attributed in part to the extremely low dry weight of the foam (about one‐half the weight of an infused composite of equivalent size and about one‐tenth the weight of a coated composite of equivalent size). The coated composites showed a swelling ratio of ≈11.0× and the infused composites showed a swelling ratio of ≈6.5×, equal to 26% and 15% of the swelling capability of the foam, respectively (Figure 3G). The swelling ratios of the composites are higher than that seen in the PVA/CMC hemostat^[^ ^32^ ^]^ and in a similar carboxymethyl chitosan/cellulose nanofiber (CMCS/CN) hemostat.^[^ ^41^ ^]^ In the case of each of these composites, increasing the presence of hydrophilic groups within the polymeric structure resulted in improved fluid uptake. It is worth noting that the coated composite was able to absorb the greatest raw mass of fluid (Figure 3H) with a 1 cm cube of sample, likely owing to the increased presence of the lyophilized nanocomposite for rehydration, while the weak structure of the diluted nanocomposite in the infused composite likely resulted in mass loss as the gelatin and nanosilicates dissolved into the PBS environment. This result supports the incorporation of the nanocomposite at the original coating formulation for advanced fluid absorption or the diluted infusion formulation for increased spread into the wound cavity.

In addition, shape recovery properties of compressed samples with and without E‐beam sterilization were analyzed in 37 °C deionized water (Figure 3I). There was not any significant difference in expansion profiles between samples treated with or without E‐beam sterilization. All the crimped samples expanded to their full diameters within three minutes. This indicates that E‐beam sterilization does not affect the shape recovery properties of the foam or the composite and promotes future clinical application. The infused composite was not tested as the foam and the coated composite represent the extremes of the designed materials.

There are some limitations in the expansion force measurement method. One assumption was that the crimped or compressed composite particle fits tightly within the wound cavity. However, this is unlikely when injecting the hemostat particles into a real wound. Therefore, the actual expansion pressure is likely to be lower than the obtained pressure values. Specific to the radial expansion force measurement, the parallel plate method only measures uniaxial force against the parallel plates along the dimension of a diameter.^[^ ^38^ ^]^ Kim et al. had shown that the radial force of coronary stents measured with this method was 7–11% of the radial force measured circumferentially.^[^ ^38^ ^]^ This indicates that the actual radial force of the composite device will be closer to 9–22 N. This is 25–65% of the radial force of commercially available coronary stents. The expected expansion pressure of the composite material, assuming it is tightly fit to the wound, will be 140–350 kPa, which is much below the minimum aneurysm wall breaking stresses of 700 kPa,^[^ ^42^ ^]^ but above typical blood pressures in human patients. This supports the conclusion that the composite is unlikely to damage tissue during or resulting from expansion. Further, as seen with the infused composites, the presence of hydrogel within the foam structure reduces the expansion force and could help in further tuning the expansion pressure.

### 2.4 Coated Composites Reduced Clotting Time by ≈20% and Infused Composites Promoted Diffuse Clotting

**Figure 4:**
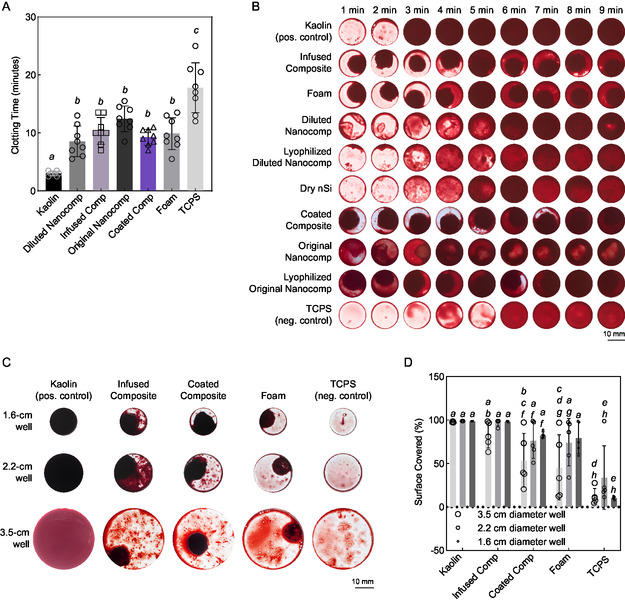
Hemostatic properties of composites. A) Quantitative clotting time measurement via inversion test (*n* = 8); bars show mean ± standard deviation (ordinary one‐way ANOVA with Tukey multiple comparisons test, different letters indicate statistically significant differences of at least *p* < 0.0332, GraphPad Prism 9, exact *p* values are shown in Table S2, Supporting Information). B) Qualitative hemostatic assessment under static conditions for composites and component materials. Representative images are shown. C) Qualitative hemostatic assessment under static conditions with variable “cavity” volumes. Representative images are shown. D) Quantification of spread of clot formation (*n* = 5); bars show mean ± standard deviation (two‐way ANOVA with Tukey multiple comparisons test, different letters indicate statistically significant differences of at least *p* < 0.0332, GraphPad Prism 9, exact *p* values are shown in Table S3, Supporting Information).

We demonstrated the potential of both infused and coated composites to promote blood clotting using both dynamic and static models. None of these models utilized the expansion capability of the composites and relied solely on contact between blood and the sample to promote clotting. The whole blood clotting time was evaluated using an inversion test (**Figure** **4**A), with kaolin as a clinically relevant positive control. Although kaolin demonstrated much faster clotting time compared to the composites, kaolin is nonbiodegradable, and so the biodegradability of the composite hemostat affords better biocompatibility and use in prolonged care scenarios. The inversion test demonstrated that the infused composite did not improve the clotting time relative to foam, which was also observed in a static assessment. In a static model with constant cavity and blood volumes, significant clotting was observed from the 5 min interval in the coated composites, infused composites, and the foam, whereas tissue culture polystyrene (TCPS) control did not show notable clotting until the 6 min mark (Figure 4B). In a second static model with variable cavity volumes, constant blood volume depth, and constant incubation time, significant spread of blood clotting was observed for the infused composite, while the coated composite and foam facilitated clotting only in their immediate surroundings (Figure 4C,D). The infused composite demonstrated diffusion of particulate matter similar to dry powder kaolin, which was not observed for the coated composite or the foam. This result is likely due to the weakened ionic interactions of gelatin and nanosilicates resulting from lower total mass percentage in the infused formulation and a subsequent ability to release hemostatic particles into the well. These results are consistent with our earlier studies, where higher percentages of nanosilicates were necessary to enhance the hemostatic potential of the system.^[^ ^11^, ^14^ ^]^ Similar results have also been observed in other novel hemostats, including CMCS/CN, of which the authors commented that the hemostatic efficacy was primarily reliant on the composites’ ability to rapidly sequester blood within a porous structure.^[^ ^40^, ^41^ ^]^

### 2.5 Composite Hemostat Demonstrates High Shape Recovery and Blood Absorption

**Figure 5:**
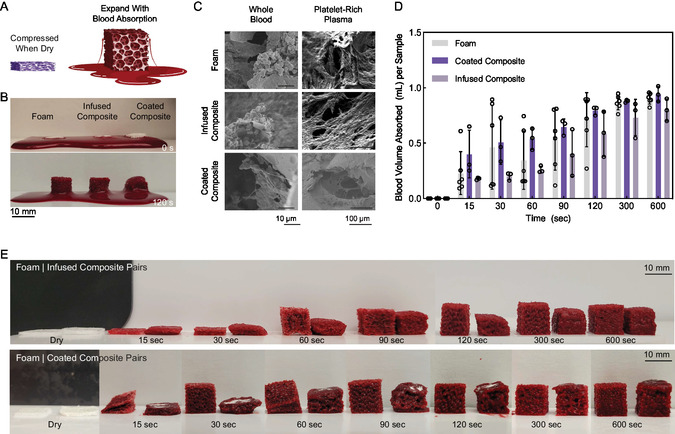
Absorption of blood by expandable composite hemostat. A) Schematic of composite expansion and blood absorption. The figure was produced using Biorender.com. B) Time‐lapse snapshots of foam, infused composite, and coated composite when placed into pooled blood at 0 s (top) and 120 s (bottom). C) SEM images showing blood cell infiltration and formation of fibrin networks within foam and composites’ porous structures. Representative images are shown. D) Quantification of blood volume absorbed by foam, infused composite, and coated composite over a period of 10 min. Experiment conducted in two rounds: Round 1: *n* = 3 foam and *n*= 3 coated composites. Round 2: *n* = 3 foam and *n* = 3 infused composites. Points show mean ± standard deviation. Two‐way ANOVA with Tukey multiple comparisons test; no statistical significance was present between sample types at any given time point. Statistical analysis conducted in GraphPad Prism 9. Exact *p* values are shown in Table S4, Supporting Information. E) Visualization of blood volume absorbed and sample expansion over a period of 10 min. (Representative images for (D)).

Each of the composites demonstrated significant expansion when placed in contact with blood (**Figure** **5**A,B; Video S1, Supporting Information). SEM imaging (Figure 5C) also showed infiltration of blood within the porous composite hemostat structure, with images taken from the internal structure of the samples. Both the foam and composite samples presented with blood cell aggregates along the internal structure, suggesting that the increased surface variation present with the addition of the nanocomposite improves red blood cell aggregation. Additional SEM images of composite samples soaked in platelet‐rich plasma exhibited extensive fibrin network formation throughout the internal structure, indicating that the composites successfully form stable clot structures within their pores. This abundance of adhered red blood cells is similarly observed in numerous novel hemostatic materials, with increased red blood cell adhesion noted for materials with cationic groups^[^ ^40^ ^]^ such as chitosan or gelatin.

Quantification (Figure 5D) of the volume of blood absorbed by the foam, infused composite, and coated composite samples (Figure 5E) revealed that the presence of the nanocomposite within the foam scaffold does not hinder the absorptive capacity of the foam upon expansion. Further, the coated composite, with its increased nanocomposite content, absorbed more blood in the early time points (up to about 90 s). All samples were able to absorb roughly the same volume of blood by the end of 10 min. In contrast to the high fluid absorption presented in the composites’ swelling ratios, the relatively lower fluid absorption by the composites compared to foam in this case is likely due to the dissociation of nanocomposite from the structure. This overall mass loss occurred as the composite expanded, and the brittle nanocomposite structure fractured off the shape‐memory foam or adhered to pooled blood around the sample. This suggests that all samples will be useful as hemostatic materials, but that the coated composite will be the most effective in short periods of time with large volumes of free fluid as the coated composite absorbed more fluid in the initial time points prior to full expansion. It is worth noting that the samples all demonstrated obvious expansion around 90–120 s, which is consistent with the shape recovery and expansion rates exhibited in earlier studies and resulted in considerable variation for the fluid uptake at these timepoints.

### 2.6 Designing Custom Applicator for Rapid Delivery of Hemostat Composite

**Figure 6:**
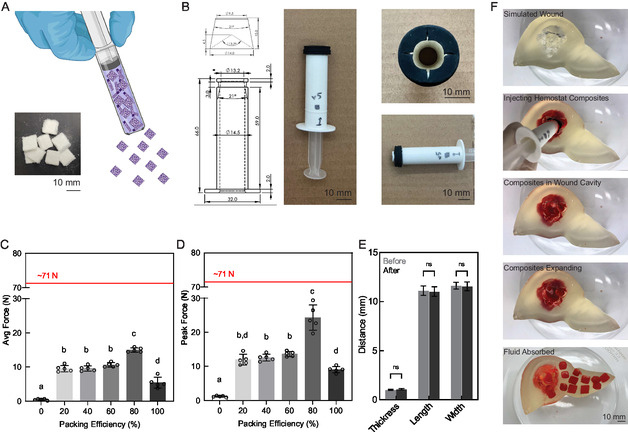
Hemostat delivery and practical use. A) Schematic of hemostatic particle injector. The figure was produced using Biorender.com. Inset: compressed infused composite particles. B) Schematics and photographs of custom‐designed injector. Schematics were produced using SolidWorks (Dassault Systèmes, France). C) Average force required to inject composite hemostat particles. *N* = 5; bars show mean ± standard deviation. Line for 71 N indicates maximum force requirement desired. Ordinary one‐way ANOVA with Tukey multiple comparisons test, different letters indicate statistically significant differences of at least *p* < 0.0332. Statistical analysis conducted in GraphPad Prism 9. Exact *p* values are shown in Table S5, Supporting Information. D) Peak force required to inject composite hemostat particles. *N* = 5; bars show mean ± standard deviation. Line for 71 N indicates maximum force requirement desired. Ordinary one‐way ANOVA with Tukey multiple comparisons test, different letters indicate statistically significant differences of at least *p* < 0.0332. Statistical analysis conducted in GraphPad Prism 9. Exact *p* values are shown in Table S6, Supporting Information. E) Composite dimensions before and after injection. *N* = 10; bars show mean ± standard deviation. Individual points are omitted for visual clarity. Two‐way ANOVA with Sidak multiple comparisons test; no statistical significance was found between measurements taken before and after injection for any given dimension. Statistical analysis conducted in GraphPad Prism 9. Exact *p* values are shown in Table S7, Supporting Information. F) Cavity model of penetrating trauma with expansion of injected particles.

In anticipation of further development of the composites as a hemostatic material, we explored a delivery mechanism for the compressed composite particles (**Figure** **6**A) with a 1 cm cube primary shape. To this end, a syringe‐like injector with a large‐bore opening was designed in SolidWorks and 3D printed (Figure 6B). We evaluated this injector device for packing density, force required to expel the composites from the injector, and any observed damage to the composites. An injector of ≈ 8 mL volume could hold 50 hemostat particles compressed to a thickness of ≈1.5 mm. Because of the injector diameter and the characteristic size of the composite particles, the 8 mL applicator facilitated neat stacking of the hemostatic particles, resulting in a packing density of ≈75% at 100% efficiency. The injection force remained well below 71 N (Figure 6C,D), which is the average strength of a thumb‐and‐two‐finger pinch typical of syringe administration.^[^ ^43^ ^]^ The dimensions of the composites did not change notably following injection, indicating that the injection mechanism did not deform the particles (Figure 6E). Finally, we tested the applicator in a custom‐designed liver defect benchtop model (Figure 6F). This model presented an irregular defect within a tissue‐like cavity. The ≈15 mL cavity was able to hold 14 particles, representing a ≈14% density at the time of injection and ≈93% density after expansion of the particles. Visual monitoring of the cavity revealed no obvious deformation following particle expansion. Overall, the custom applicator can rapidly expel hemostatic particles into a deep wound cavity, thus supporting the use of this composite formulation to resolve hemorrhage in emergency situations.

## 3 Conclusion

Noncompressible hemorrhage presents a significant risk to life and is difficult to manage without advanced interventions, namely surgery. Further, many current solutions are not biodegradable and require extended surgical times to remove the hemostatic product. A potential solution to these problems is a biomaterial capable of rapidly expanding within a wound cavity, exerting a compressive force on ruptured vessels, reducing clotting time, and exhibiting cytocompatible degradation. Here, we describe the fabrication and evaluation of nanocomposite‐foam structures to achieve these desirable properties. The combination of these materials capitalizes on the well‐characterized hemostatic ability of a gelatin‐nanosilicate nanocomposite and expansion property of a shape‐memory foam, thus overcoming the limitations of nonexpanding hemostatic biomaterials. In addition, we demonstrate the ability to tune these mechanical and hemostatic properties based on nanocomposite formulation. The coated composite in particular demonstrates increased expansion force and decreased clotting time compared to the foam alone, supporting the use of this material in noncompressible wounds. Similarly, the infused composite exhibits an increased clotting influence compared to either the coated composite or foam, illuminating its potential for large‐volume wounds. Overall, these results signify the capability of this composite material to rapidly achieve hemostasis in noncompressible wound cavity with improvements over current treatment options. Future work will continue to evaluate the ability of this composite material to control severe, noncompressible hemorrhage in terms of biodistribution, long‐term biodegradation, and ability to control hemorrhage in physiological models.

## 4 Experimental Section

4.1

4.1.1

##### Shape‐Memory Foam Synthesis

Shape‐memory polyurethane foams were synthesized using the three‐step foaming process as described previously.^[^ ^21^ ^]^ The H40 foam composition (“foam”) was chosen due to its fast shape recovery rate compared to other shape‐memory foam compositions.^[^ ^15^ ^]^ First, an isocyanate (NCO) premix was made in a desiccated glove box by mixing 40 equivalents of hydroxyl groups from N,N,N′,N′‐tetrakis(2‐hydroxypropyl) ethylenediamine (HPED; Sigma–Aldrich, USA), 60 equivalents of hydroxyl groups from TEA (98% Alfa Aesar Inc., USA), and 100 equivalents of isocyanate groups from HDI (TCI Chemicals, USA). The NCO premix was thoroughly mixed and cured for 2 days in an oven with a preset cure cycle (ramp rate 20 °C h^−1^, held at 50 °C for 36 h). Secondly, a hydroxyl (OH) premix was prepared by mixing the balance of hydroxyl groups from HPED, TEA, and deionized water using a FlackTek Speedmixer at 3400 rpm for 30 s. For the final step, the NCO premix and the OH premix were combined along with surfactants (DC 198, DC5943; 3400 rpm, 30 s; Evonik, USA), catalysts (T‐131, BL‐22; 3400 rpm, 30 s; Evonik, USA), and a physical blowing agent (Enovate; 3400 rpm, 5 s; Evonik, USA). The mixture was allowed to foam at 50 °C for 3–6 min. The detailed amounts of components in the final foam are summarized in **Table** **1**.

**Table 1:** H40 foam composition.

Sample ID	HDI [wt%]	HPED [wt%]	TEA [wt%]	Water [wt%]	DC198 [wt%]	DC5943[wt%]	T‐131 [wt%]	BL‐22 [wt%]	Enovate[pph]
H40	62.8	13.1	13.4	2.7	4.0	3.0	0.03	0.6	5.0

##### Composite Fabrication

In order to determine the total mass percentage which could effectively permeate the foam's porous structure, a concentration sweep of both nanosilicates (Laponite XLG; BYK, USA) and gelatin (Type A, 300 Bloom strength; Sigma–Aldrich, USA) was performed and the infusion depth of the nanocomposite into 1 cm cubes of polyurethane foam was evaluated. Concentrations ranging from 1.5 to 6% nanosilicates and 0.75–3% gelatin were synthesized and the composites were prepared according to the procedure outlined below. These nanocomposites were dyed green using food coloring for color contrast and evaluated for their ability to penetrate the entire thickness of the foam sample when incubated at 60 °C for up to 1 h. The foams were then sliced transversely, and photographs were taken at a standardized height using a smartphone camera. The penetration depth of the nanocomposite was measured across three distinct dimensions using ImageJ (NIH). Initial measurements were obtained in units of pixels and were then converted to millimeters based on an average of 5 measurements taken for a known distance. This unit conversion was performed in Microsoft Excel and data in units of millimeters was subsequently plotted and analyzed. Based on the results from this analysis and previous work optimizing the nanocomposite's composition for desirable hemostatic and mechanical properties,^[^ ^11^ ^]^ a coated formulation of 6% w/v nanosilicate and 3% w/v gelatin and an infused formulation of 1.5% w/v nanosilicate and 0.75% gelatin nanocomposite were selected for further analysis.

For the coated composites, foams were punched into a cylindrical shape with 1 cm diameter and 1 cm length using a biopsy punch. Nanosilicates (final concentration 6% w/v) were added to deionized water at 4 °C and allowed to exfoliate to optical clarity. Gelatin (final concentration 3% w/v) was dissolved separately in deionized water at 37 °C. The gelatin and nanosilicate solutions were combined and mixed until homogenous. Homogeneous nanocomposite was kept warm at in an oven at 60 °C. Foams were soaked in the composite for 1 h to allow the infusion of nanocomposite within the foam structure. To further facilitate infusion, the foam‐nanocomposite structure was placed in a vacuum chamber for 2 h. Then, the structures were flash frozen using liquid nitrogen or stored at −80 °C overnight and then lyophilized overnight. Foams and composites were stored in a desiccated vacuum chamber until use. Fabrication of cubes and discs occurred in the same manner.

Because the coated nanocomposite formulation struggled to infiltrate the foam's porous structure, the foam used to fabricate the coated composites was mechanically reticulated according to a previous procedure.^[^ ^20^ ^]^ Reticulation was performed to ensure open‐pore structure of the foams which would enhance nanocomposite penetration, blood clotting, cell migration, and wound healing process. In brief, the synthesized foam was mounted on an oscillating vibratory shaker. A gravity‐driven floating nitinol pin array (50 mm length) was loaded on top of the foam perpendicularly with the tips of unrestricted nitinol pins barely touching the top surface of the foam. The foam was agitated by the shaker while the floating pins moved downward penetrating the foam. The pin array moved 500 μm horizontally to punch the foam. The foam was flipped and the same process was done on the other side of the foam. Reticulated foams were sonicated in reverse osmosis (RO) water with 1:20 volume ratio for 30 min. RO water was switched to isopropyl alcohol (IPA), and samples underwent sonication four times changing the IPA to a fresh solution every 30 min. Samples were sonicated further in RO water four times changing to a fresh solution every 15 min. Cleaned foams without any residual foaming additives were dried in 50 °C oven overnight and stored at room temperature under vacuum until use.

For the infused nanocomposites, foams were again punched into cylinders (1 cm diameter, 1 cm length). Nanosilicates (final concentration 1.5% w/v) were added to deionized water at 4 °C and allowed to exfoliate to optical clarity. Gelatin (final concentration 0.75% w/v) was dissolved in deionized water at 37 °C and mixed until homogeneous. This infused nanocomposite composition was heated to 60 °C and foams were soaked in the nanocomposite for 1 h to allow infusion within the foam structure. To further facilitate infusion, the composite was placed in a vacuum chamber for 2 h. Then, the composites were frozen at −80 °C overnight and subsequently lyophilized. Foams and composites were stored at room temperature in a desiccated vacuum chamber until use. Fabrication of cubes and discs occurred in the same manner.

##### Structural Characterization

Fabricated foams, gelatin‐nanosilicate nanocomposite, and both coated and infused composites were placed in microcentrifuge tubes and dipped in liquid nitrogen for ≈30 s and lyophilized. These samples were then sectioned transversely into two halves and were mounted on an adhesive carbon tape attached to a stainless‐steel stub. All samples were then sputter coated with iridium or platinum–palladium in an inert atmosphere of argon gas. Using a scanning electron microscope, surface topography for each sample was observed at 70× magnification using a 20 kV electron beam (Quanta 600 FE‐SEM, FEI, USA; Research Resource ID: SCR_022128) or at 25× magnification using a 5 kV electron beam (JSM‐7500F, JEOL, Japan; Research Resource ID: SCR_022202). Additionally, energy‐dispersive X‐ray spectroscopy (EDS) was performed to conduct an elemental analysis of the samples to detect the presence of Mg and Si in the composites.

##### Swelling and Expansion Characteristics

Furthermore, dry weight (*W*_d_) of noncompressed foam and composite samples were measured after taking them out from a desiccated vacuum chamber. They were submerged in 37 °C PBS for 24 h and weighed after removing from the PBS to get the swollen weight (*W*_s_). The swelling ratio (*Q*) was calculated using the equation below.

(1) $$Q=\frac{W_{s}}{W_{d}}$$

For expansion studies, 1 cm cubic foams and composites were preheated at 70 °C for 1 h prior to compression. The samples were placed between the platens of a hot press (Carver, USA) and allowed to equilibrate with the machine at 80 °C for 15 min. The samples were then axially compressed to a height of 1–1.5 mm and held in this secondary shape at 80 °C for another 15 min. The samples were then cooled to room temperature before releasing the platens to fix the secondary shape. After measuring the compressed heights of the samples, they were placed in 37 °C deionized water, PBS, or citrated bovine plasma where the expanding height of each sample was measured at specified time intervals (0, 10, 30, 60, 90, 120, and 180 s). All the heights were measured using ImageJ by measuring the height at three evenly spaced locations along the sample length. Sample volume fold change was calculated by the following equation

(2) $$\text{Volume fold change}=\left(\frac{\text{Expanded sample height}}{\text{Initial compressed height}}\right)$$

##### Expansion Force Measurement

Expansion force measurement in a physiological environment was subsequently measured using an RSA‐G2 dynamic mechanical analysis instrument (TA Instruments, USA). The sample was placed between two compression plates within an immersion chamber. The plates remained static throughout the experiment. The immersion chamber was filled with 37 °C PBS to submerge both the sample and the plates. A forced‐convection oven was used to maintain the temperature around the immersion chamber and sample. The force of the compressed sample while expanding axially was measured within the temperature range of 35–37 °C. A blank test was conducted with no sample to determine the buoyancy force due to the presence of the PBS. This force was subtracted out from the raw force values determined for each sample to calculate the force due to axial expansion.

For radial expansion force measurement, the cylindrical foams and the coated composites were radially crimped by placing the samples inside a heated ST 150‐42 stent crimper (Machine Solutions, USA) at 70 °C for 5 min to allow the heat equilibration throughout the samples. Then, the samples were radially crimped to a diameter of 2–2.5 mm and immediately cooled down to room temperature to fix the secondary shape. The crimped samples were stored in a desiccated vacuum chamber at least for 24 h before use. The crimped sample was placed between two compression platens inside a waterproof environmental chamber which was attached to the Instron Model 5966 Dual Column Test System (Illinois Tool Works Inc., USA). After the sample was given a preload of 0.15 N, 40 °C RO water was filled into the water chamber up to the height which can submerge both platens and the sample. The expansion forces of the crimped samples were measured within the temperature range of 36.5–40 °C and the two compression platens remained still throughout the test. This analysis method is based on ISO 25539‐2. The buoyancy force of water was measured by performing the same test without the sample between the platens. The expansion force was calculated by subtracting the buoyancy force from the raw force recorded.

##### Degradation Studies

Both hydrolytic and oxidative accelerated degradation studies were conducted. For all studies, the initial masses of the samples were measured and recorded. For the hydrolytic degradation study, only the foam and the coated composite were analyzed as extremes of the fabrications. In this study, the samples were submerged in 0.1 N NaOH at a 1:20 volume ratio (sample: NaOH solution) in individual glass vials. The samples were stored in a 37 °C incubator and the solutions were exchanged to fresh solution every 3 days to keep constant pH and ion concentration. The sample masses were measured every 6 days after draining the sample solution, washing with RO water and IPA, and drying overnight under vacuum at 50 °C. Because the mass remained relatively constant for both samples over a period of 90 days, we elected to end the hydrolytic analysis here.

For the oxidative degradation study, all three sample types (foam, infused composite, and coated composite) were examined. Again, all samples were weighed prior to the study start. Samples were submerged in 20% hydrogen peroxide (H_2_O_2_) solution at a 1:20 volume ratio in individual glass vials. The samples were stored in a 37 °C incubator and the solutions were exchanged for fresh ones every 3 days to keep constant pH and ion concentration. The sample weights were measured every 6 days after draining the sample solution, washing with deionized water, and freeze drying the samples for 48 h. For degraded foam analysis, ATR‐FTIR spectroscopy (ALPHA Infrared Spectrometer with a diamond ATR crystal, Bruker, USA) and SEM imaging (JSM‐7500F, JEOL, Japan; Research Resource ID: SCR_022202) were used to characterize foam chemistry and foam morphologies at the initial time point and at 30 days. Bruker OPUS Spectroscopy software (Bruker, USA) was used to analyze the obtained data. A second oxidative degradation study was conducted out to 90 days with only the foam and coated composites as extremes of the fabrication conditions. This study was conducted using the protocols described earlier.

##### Hemostatic Assessments

The composites were assessed for their hemostatic properties, both in terms of blood absorption and reduction of the clotting time, using bovine blood containing CPDA‐1 (citrate‐phosphate‐dextrose solution with adenine) anticoagulant collected no more than 3 weeks prior to experimentation. Throughout the experiments, kaolin was used as a positive control to ensure that blood coagulation was still within normal ranges. The hemostatic ability of the composites was tested in both static and dynamic conditions along with imaging to assess the infiltration of blood within the porous structure.

For static conditions, composite samples were placed in a well plate and prewarmed whole bovine blood at 37 °C was added to each well. The blood was recalcified with 0.1 m calcium chloride (VWR, USA) in a 9:1 volumetric ratio to inactivate the anticoagulant immediately prior to testing. At each 1 min interval, unclotted blood was removed from the well. The samples were then imaged using a stereomicroscope (Zeiss Discovery V8, Germany) to visualize the clot formed at each interval. TCPS (negative control), foams, nanocomposite formulations, and kaolin (positive control) were used as controls. A second static condition was conducted similarly to the aforementioned experiment, with the modification that various‐sized well plates were used to assess the impact of clotting over variable distances and at a constant time interval. The volume of the blood added to the well was modulated to maintain a constant submersion depth for the sample. The wells were imaged using a stereomicroscope to visualize the diffusion of particulate matter and clot formation throughout the wells.

For the dynamic phase model, the clotting time was measured using an inversion test. Samples and recalcified bovine blood (9 parts whole blood: 1 part 0.1 m CaCl_2_) were added to a well in a 24‐well tissue culture plate. The plate was incubated at 37 °C and inverted to 90° every 30 s to check for clot formation. Clotting time was determined when no visible blood flow was present. TCPS served as a negative control; kaolin served as a positive control.

##### Blood Absorption Evaluation

The fluid uptake of the composite samples was assessed to monitor expansion in physiological conditions. Foam and composite samples were prepared and axially compressed as previously described. The samples were each weighed prior to testing. Samples were placed into a well of preheated, citrated bovine blood and allowed to expand for predetermined time intervals (15–600 s). After the specified time, samples were removed from the blood and weighed. A calibration curve for blood density was determined to convert raw mass values into blood volume absorbed.

Samples were also imaged using SEM to evaluate the infiltration of blood cells within the porous structure of the composite samples. Composite and foam samples were prepared and compressed axially as previously described. Samples were placed into recalcified bovine blood at 37 °C and allowed to expand for 10 min. Samples were then removed from the blood and fixed for 24 h with 4% paraformaldehyde (Fisher Scientific, USA). Samples were dehydrated in a graded ethanol series followed by immersion in hexamethyldisilane and allowed to air dry. Samples were then sputter coated with platinum–palladium coating and imaged. This experiment was repeated with samples immersed in platelet‐rich plasma to better visualize fibrin clot formation and platelet activation without the presence of red blood cells. Platelet activation was not observed due to the dense fibrin network formation.

##### Sterilization and Cytocompatibility

Samples were sterilized with E‐beam radiation at the National Center for Electron Beam Research (College Station, TX). Cylindrical samples and humidity indicators (S‐8028 10–60% Humidity Indicator, Uline, USA) were packaged in foil header pouches (Tyvek Foil Pouches, Beacon Converters, USA) using an AVN packaging system (AmeriVacS, USA). Nitrogen gas was purged into the pouch right before it was vacuum sealed with heat. E‐beam dose of 43.8 kGy was radiated using a vertically mounted 10 MeV, 18 kW commercial scale linear accelerator. Alanine films (Kodak, USA) were placed below samples to measure the actual absorbed radiation dose using a Bruker E‐scan spectrometer (Bruker, USA). E‐beam sterilized samples were chemically characterized using ATR‐FTIR and compared to the nonsterilized samples. These samples were also evaluated in terms of expansion ability to ensure that the sterilization process did not disrupt the mechanical properties. Cylindrical samples were crimped as described previously. After measuring the crimped diameters of the samples, they were immersed in 37 °C deionized water, where the expanding diameters of each sample were measured (every 30 s up to 5 min, every 1 min over 5–10 min). All the diameters were measured using ImageJ (NIH) by measuring the diameter at three evenly spaced locations along the sample length. Diameter recovery (%) was calculated by the following equation.

(3) $$\text{Diameter recovery}\left(\%\right)=\left(\frac{\text{Recovered diameter}}{\text{Initial diameter}}\right)\times 100\%$$

Sterilization was also performed using exposure to UV light exposure as a method for decreasing costs and improving accessibility of sterilization procedures. The samples were exposed to UV light at 365 nm and a distance of ≈8 cm for 10 min per side. Samples were rotated every 10 min to ensure complete coverage and facilitate penetration of light into all areas of the sample.

The samples were subsequently evaluated for their impact on cellular viability and proliferation using an indirect contact study. Two coated composite samples (total mass ≈ 40 mg) were sterilized and placed into 40 mL of alpha‐modified Eagle's media supplemented with 10% FBS and 1% penicillin/streptomycin (AMEM). After 48 h, the composites were removed. The resulting media was termed “leached media” for containing leachables from the composites. Human mesenchymal stem cells (hMSCs) were cultured in a well plate at 37 °C under 5% CO_2_. Upon reaching 75% confluency, the cells were dosed with the leached media at varying concentrations ranging from 0 to 100% leached media. Media was changed every day to maintain constant exposure to the leachables. An alamarBlue assay was performed on days 1, 3, 5, and 7 to determine the toxicity of the leachables from the composites at each time point. Absolute percentage reduction of alamarBlue was calculated using the following formula described by BioRad:

(4) $$\text{Percentage reduction of alamarBlue}=\frac{\left(O2\times A1)-(O1\times A2\right)}{\left(R1\times N2)-(R2\times N1\right)}\;\times \;100\%$$

where

*O*1 = molar extinction coefficient of oxidized alamarBlue at 570 nm

*O*2 = molar extinction coefficient of oxidized alamarBlue at 600 nm

*R*1 = molar extinction coefficient of reduced alamarBlue at 570 nm

*R*2 = molar extinction coefficient of reduced alamarBlue at 600 nm

*A*1 = absorbance of test wells at 570 nm

*A*2 = absorbance of test wells at 600 nm

*N*1 = absorbance of negative control well (media plus alamarBlue but no cells) at 570 nm

*N*2 = absorbance of negative control well (media plus alamarBlue but no cells) at 600 nm.

The absolute percentage reduction of alamarBlue was normalized to the absolute percentage reduction of alamarBlue from day 1 for each sample. This result was termed “cellular proliferation.”

##### Injection Force Testing and Practical Use Proof‐of‐Concept

A custom, large‐bore injector was designed in SolidWorks (Dassault Systèmes, France) and 3D printed (Ender‐3 Pro; Creality, China) using polylactic acid (PLA) filament (Hatchbox, USA). An injector volume of ≈8 mL was selected for intended use in small animals. Compressed composite particles were loaded into the injector in a neat, stacked orientation. Packing density was calculated as the volume of the particles divided by the volume of the injector lumen.

(5) $$\text{Packing density}\left(\%\right)=\left(\frac{\text{Volume occupied by composites}}{\text{Injector inner volume}}\right)\times 100\%$$

Packing efficiency was determined by loading varying numbers of particles (i.e., variable volumes of particles) into the injector and expressed as a percentage of the maximum packing density. The packing efficiency was varied from 0 to 100% to explore the impact of packing efficiency on the force required to eject the particles. This force was measured using a mechanical testing machine (MTest QUATTRO, ADMET, USA). The injector plunger was depressed at a rate of 1.5 cm s^−1^, equating to 3.5 mL s^−1^ which would empty the injector within 5 s. The test was set to run until the machine measured 50 N or fully emptied the injector. The ejector was fully emptied in all tests. The dimensions of the composite particles were measured before and after the injection process to determine if the injection caused deformation to the particles.

A benchtop liver wound model was fabricated to evaluate the practical use of the designed injector and the ability of the composite hemostat to address bleeding in a wound cavity. An irregular defect of ≈15 mL was created in a block of 10% gelatin (300 Bloom, Type A). This cavity was filled with PBS (dyed red) which was preheated such that the temperature of the PBS present in the cavity remained at ≈37 °C. Compressed composites were injected into the cavity using the designed injector and allowed to expand. After 4 min, composites were removed from the cavity to observe fluid absorption and any gross deformation to the cavity.

##### Statistical Analysis

All statistical analysis was conducted using GraphPad Prism 9. Outliers were neither identified nor removed. Any preprocessing of data was performed in Microsoft Excel. Preprocessing was limited to normalization unless otherwise indicated in the figure caption. FTIR data was shifted in the *y* direction in order to display data as stacked spectra rather than overlaid spectra; all data for a given sample was shifted by the same value. Multiple comparisons were performed using an ordinary one‐way ANOVA with Tukey's post hoc test. Grouped analyses were performed using a two‐way ANOVA with Tukey's multiple comparisons tests unless otherwise noted based on data categories. Individual points are shown to indicate sample size unless otherwise noted. Bars show mean ± standard deviation (for one reading on multiple replicates) or mean ± standard error of the mean (for multiple readings on each of multiple replicates) as indicated. Statistical significance is indicated by *p < 0.0332, **p < 0.0021, ***p < 0.0002, and ****p < 0.0001.

## Conflict of Interest

The shape memory foam in this paper is licensed by Shape Memory Medical Inc., and the hydrogel in this paper is licensed by Boston Scientific. D.J.M discloses an active role as a Director in Shape Memory Medical Inc. and holds both shares and stock options. Neither Shape Memory Medical Inc. nor Boston Scientific funded this work.

## Author Contributions

**Sarah E. Hargett**: conceptualization (lead); data curation (lead); formal analysis (lead); visualization (lead); writing—original draft (lead). **Giriraj K. Lokhande**: conceptualization (lead); data curation (lead); formal analysis (lead); visualization (lead); writing—original draft (lead). **Joseph Duran**: data curation (supporting); formal analysis (supporting). **Zanir Hirani**: investigation (supporting); methodology (supporting). **Lindy K. Jang**: investigation (supporting); methodology (supporting). **Samantha Foster**: formal analysis (supporting); investigation (supporting). **Kaivalya A. Deo**: investigation (supporting); methodology (supporting). **Sasha George**: investigation (supporting); methodology (supporting). **Mahjabeen Javed**: investigation (supporting); methodology (supporting). **Taylor H. Ware**: investigation (supporting); resources (supporting); supervision (supporting). **Duncan J. Maitland**: conceptualization (lead); funding acquisition (lead); supervision (lead); writing—original draft (lead). **Akhilesh K. Gaharwar**: conceptualization (lead); data curation (lead); formal analysis (lead); funding acquisition (lead); supervision (lead); visualization (lead); writing—original draft (lead). **Sarah E. Hargett** and **Giriraj K. Lokhande** contributed equally to this work.

## Supporting information

Supplementary Material

## Data Availability

The data that support the findings of this study are available in an online Zenodo repository at the following DOI: 10.5281/zenodo.13982589.
