# Supplementary material for: Nanoengineered Shape‐Memory Hemostat
Source: Small Sci. 2024 Dec 11;5(2):2400321. doi: 10.1002/smsc.202400321 (PMC11934902; doi:10.1002/smsc.202400321)
Supplement: Supplementary file 1 — Supplementary Material [file SMSC-5-2400321-s001.zip › smsc202400321-sup-0001-SuppData-S1.pdf]

# Supporting Information

**Nanoengineered Shape-memory Hemostat**

*Sarah E. Hargett[1,#], Giriraj K. Lokhande[1,#], Joseph Duran[1], Zanir Hirani[1], Lindy K. Jang[1,5], Samantha Foster[3], Kaivalya A. Deo[1], Sasha George[2], Mahjabeen Javed[1], Taylor H. Ware[1,2], Duncan J. Maitland[1,*], and Akhilesh K. Gaharwar[1,2,3,4,*]*

[1]Department of Biomedical Engineering, College of Engineering, Texas A&M University, College Station, TX 77843, USA

[2]Department of Material Science and Engineering, College of Engineering, Texas A&M University, College Station, TX 77843, USA.

[3]Interdisciplinary Program in Genetics, Texas A&M University, College Station, TX 77843, USA

[4]Center for Remote Health Technologies and Systems, Texas A&M University, College Station, TX 77843, USA.

[5]Materials Engineering Division, Lawrence Livermore National Laboratory, Livermore, CA 94550, USA.

[#]Equally contributed

*Corresponding author E-mail: gaharwar@tamu.edu (AKG); djmaitland@tamu.edu (DJM)

Keywords: expandable biomaterials, hemostats, nanocomposite, wound healing, porous materials

**Table S1.** One-way ANOVA probability values for Figure 1B.

| Tukey's multiple comparisons test | Mean Diff. | 95.00% CI of diff. | Below threshold? | Summary | Adjusted P Value |
|---|---|---|---|---|---|
| 6% nSi 3% gel vs. 3% nSi 1.5% gel | -0.3883 | -2.556 to 1.780 | No | ns | 0.9578201904 |
| 6% nSi 3% gel vs. 2% nSi 1% gel | -1.302 | -3.470 to 0.8661 | No | ns | 0.3592475045 |
| 6% nSi 3% gel vs. 1.5% nSi 0.75% gel | -8.564 | -10.73 to -6.396 | Yes | **** | 0.0000000033 |
| 3% nSi 1.5% gel vs. 2% nSi 1% gel | -0.9136 | -3.081 to 1.254 | No | ns | 0.6462431822 |
| 3% nSi 1.5% gel vs. 1.5% nSi 0.75% gel | -8.176 | -10.34 to -6.008 | Yes | **** | 0.0000000073 |
| 2% nSi 1% gel vs. 1.5% nSi 0.75% gel | -7.262 | -9.430 to -5.095 | Yes | **** | 0.0000000528 |

Data analysis is conducted in GraphPad Prism 9. Ordinary one-way ANOVA with Tukey's multiple comparisons post-hoc analysis. P*<0.0332, P**<0.0021, P***<0.0002, P****<0.0001.

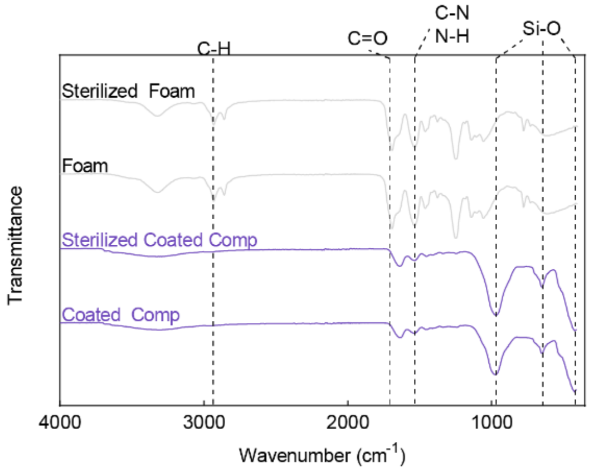


**Figure S1.** FTIR spectra of foam and coated composite before and after ultraviolet (UV) light exposure sterilization.

Samples were exposed to UV light at 365 nm for 1 hour. No changes are noted in the peaks between the sterilized and non-sterilized samples, indicating that this method for sterilization does not produce chemical changes in either the form or the composite.

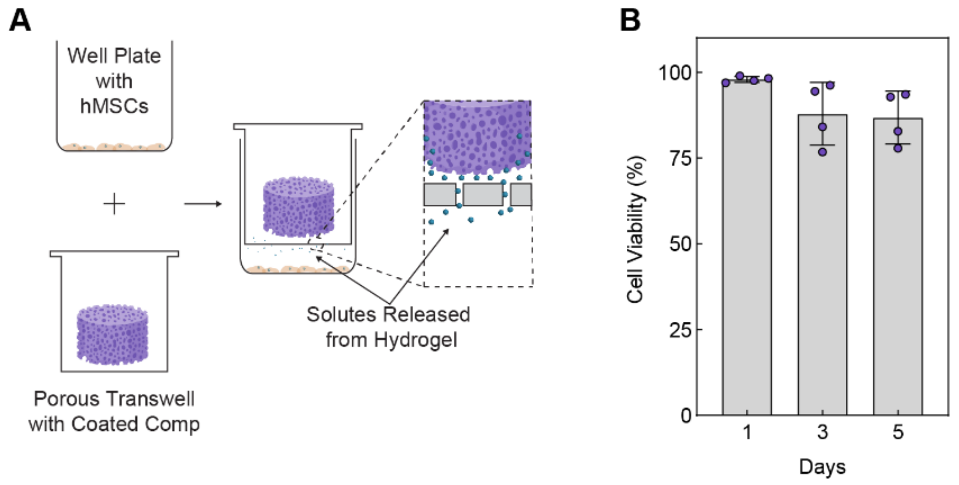


**Figure S2.** Transwell assay of cell viability with coated composite.

S2A) Schematic showing experimental setup for transwell assay, wherein cells are indirectly exposed to the composite through release of leached solutes into cell culture media.

S2B) Cellular viability normalized to control (i.e., untreated) cells.

The sterilized samples were additionally tested in a transwell assay **(Figure S2A)** to determine biocompatibility. The use of transwells allowed for indirect contact between the composites and cells. We observed weight loss from the coated composites as seen in earlier experiments, with accumulation of analytes in media. We speculate $Mg^{2+}$ and Si ions from the composite surface could be introduced into the medium supplementing hMSC growth. With this leaching of analytes, hMSCs showed >90% viability when normalized to untreated TCPS controls for each day **(Figure S2B)**. There was no statistical significance between the viability on day 1, 3 and 5 thus demonstrating that the leachables generated after incubation with composite foams did not affect the survival of cells.

**Table S2.** One-way ANOVA probability values for Figure 4B.

| Tukey's multiple comparisons test | Mean Diff. | 95.00% CI of diff. | Below Threshold? | Summary | Adjusted P Value |
|---|---|---|---|---|---|
| Kaolin vs. Orig. Nanocomposite | -9.438 | -13.35 to -5.521 | Yes | **** | <0.0001 |
| Kaolin vs. Coated Comp | -6.250 | -10.17 to -2.333 | Yes | *** | 0.0002 |
| Kaolin vs. Foam | -6.875 | -10.79 to -2.958 | Yes | **** | <0.0001 |
| Kaolin vs. Dil. Nanocomposite | -5.563 | -9.479 to -1.646 | Yes | ** | 0.0012 |
| Kaolin vs. Infused Comp | -7.375 | -11.29 to -3.458 | Yes | **** | <0.0001 |
| Kaolin vs. TCPS | -14.81 | -18.73 to -10.90 | Yes | **** | <0.0001 |
| Orig. Nanocomposite vs. Coated Comp | 3.188 | -0.7290 to 7.104 | No | ns | 0.1812 |
| Orig. Nanocomposite vs. Foam | 2.563 | -1.354 to 6.479 | No | ns | 0.4209 |
| Orig. Nanocomposite vs. Dil. Nanocomposite | 3.875 | -0.04153 to 7.792 | No | ns | 0.0542 |
| Orig. Nanocomposite vs. Infused Comp | 2.063 | -1.854 to 5.979 | No | ns | 0.6711 |
| Orig. Nanocomposite vs. TCPS | -5.375 | -9.292 to -1.458 | Yes | ** | 0.0019 |
| Coated Comp vs. Foam | -0.6250 | -4.542 to 3.292 | No | ns | 0.9989 |
| Coated Comp vs. Dil. Nanocomposite | 0.6875 | -3.229 to 4.604 | No | ns | 0.9981 |
| Coated Comp vs. Infused Comp | -1.125 | -5.042 to 2.792 | No | ns | 0.9735 |
| Coated Comp vs. TCPS | -8.563 | -12.48 to -4.646 | Yes | **** | <0.0001 |
| Foam vs. Dil. Nanocomposite | 1.313 | -2.604 to 5.229 | No | ns | 0.9444 |
| Foam vs. Infused Comp | -0.5000 | -4.417 to 3.417 | No | ns | 0.9997 |
| Foam vs. TCPS | -7.938 | -11.85 to -4.021 | Yes | **** | <0.0001 |
| Dil. Nanocomposite vs. Infused Comp | -1.813 | -5.729 to 2.104 | No | ns | 0.7871 |
| Dil. Nanocomposite vs. TCPS | -9.250 | -13.17 to -5.333 | Yes | **** | <0.0001 |
| Infused Comp vs. TCPS | -7.438 | -11.35 to -3.521 | Yes | **** | <0.0001 |

**Table S3**. Two-way ANOVA probability values for Figure 4E.

| Tukey's multiple comparisons test | Mean Diff. | 95.00% CI of diff. | Below threshold? | Summary | Adjusted P Value |
|---|---|---|---|---|---|
| | | | | | |
| Kaolin | | | | | |
| 6 well vs. 12 well | -0.9660 | -30.08 to 28.15 | No | ns | 0.9965 |
| 6 well vs. 24 well | -0.9370 | -30.05 to 28.18 | No | ns | 0.9967 |
| 12 well vs. 24 well | 0.02903 | -29.09 to 29.15 | No | ns | >0.9999 |
| Infused Comp | | | | | |
| 6 well vs. 12 well | -14.28 | -43.39 to 14.84 | No | ns | 0.4706 |
| 6 well vs. 24 well | -15.65 | -44.77 to 13.47 | No | ns | 0.4053 |
| 12 well vs. 24 well | -1.375 | -30.49 to 27.74 | No | ns | 0.9929 |
| Coated Comp | | | | | |
| 6 well vs. 12 well | -22.99 | -52.10 to 6.130 | No | ns | 0.1483 |
| 6 well vs. 24 well | -29.11 | -58.23 to 0.004354 | No | ns | 0.0500 |
| 12 well vs. 24 well | -6.126 | -35.24 to 22.99 | No | ns | 0.8689 |
| Foam | | | | | |
| 6 well vs. 12 well | -28.91 | -58.03 to 0.2051 | No | ns | 0.0520 |
| 6 well vs. 24 well | -33.98 | -63.10 to -4.863 | Yes | * | 0.0183 |
| 12 well vs. 24 well | -5.068 | -34.18 to 24.05 | No | ns | 0.9082 |
| TCPS | | | | | |
| 6 well vs. 12 well | -21.58 | -50.69 to 7.538 | No | ns | 0.1846 |
| 6 well vs. 24 well | 1.720 | -27.40 to 30.84 | No | ns | 0.9890 |
| 12 well vs. 24 well | 23.30 | -5.818 to 52.41 | No | ns | 0.1410 |
| 6 well | | | | | |
| Kaolin vs. Infused Comp | 15.01 | -19.06 to 49.09 | No | ns | 0.7287 |
| Kaolin vs. Coated Comp | 44.05 | 9.973 to 78.12 | Yes | ** | 0.0050 |
| Kaolin vs. Foam | 52.29 | 18.22 to 86.36 | Yes | *** | 0.0006 |
| Kaolin vs. TCPS | 85.52 | 51.45 to 119.6 | Yes | **** | <0.0001 |
| Infused Comp vs. Coated Comp | 29.04 | -5.039 to 63.11 | No | ns | 0.1304 |
| Infused Comp vs. Foam | 37.28 | 3.204 to 71.35 | Yes | * | 0.0252 |
| Infused Comp vs. TCPS | 70.51 | 36.43 to 104.6 | Yes | **** | <0.0001 |
| Coated Comp vs. Foam | 8.243 | -25.83 to 42.32 | No | ns | 0.9600 |
| Coated Comp vs. TCPS | 41.47 | 7.399 to 75.55 | Yes | ** | 0.0095 |
| Foam vs. TCPS | 33.23 | -0.8438 to 67.30 | No | ns | 0.0593 |
| 12 well | | | | | |
| Kaolin vs. Infused Comp | 1.702 | -32.37 to 35.78 | No | ns | >0.9999 |
| Kaolin vs. Coated Comp | 22.03 | -12.05 to 56.10 | No | ns | 0.3730 |
| Kaolin vs. Foam | 24.34 | -9.729 to 58.42 | No | ns | 0.2741 |
| Kaolin vs. TCPS | 64.91 | 30.83 to 98.98 | Yes | **** | <0.0001 |
| Infused Comp vs. Coated Comp | 20.33 | -13.75 to 54.40 | No | ns | 0.4553 |
| Infused Comp vs. Foam | 22.64 | -11.43 to 56.72 | No | ns | 0.3451 |
| Infused Comp vs. TCPS | 63.21 | 29.13 to 97.28 | Yes | **** | <0.0001 |
| Coated Comp vs. Foam | 2.317 | -31.76 to 36.39 | No | ns | 0.9997 |
| Coated Comp vs. TCPS | 42.88 | 8.806 to 76.95 | Yes | ** | 0.0068 |

| | | | | | |
|---|---|---|---|---|---|
| Foam vs. TCPS | 40.56 | 6.489 to 74.64 | Yes | * | 0.0119 |
| 24 well | | | | | |
| Kaolin vs. Infused Comp | 0.2983 | -33.78 to 34.37 | No | ns | >0.9999 |
| Kaolin vs. Coated Comp | 15.87 | -18.20 to 49.95 | No | ns | 0.6861 |
| Kaolin vs. Foam | 19.25 | -14.83 to 53.32 | No | ns | 0.5104 |
| Kaolin vs. TCPS | 88.18 | 54.10 to 122.3 | Yes | **** | <0.0001 |
| Infused Comp vs. Coated Comp | 15.57 | -18.50 to 49.65 | No | ns | 0.7010 |
| Infused Comp vs. Foam | 18.95 | -15.12 to 53.02 | No | ns | 0.5259 |
| Infused Comp vs. TCPS | 87.88 | 53.80 to 122.0 | Yes | **** | <0.0001 |
| Coated Comp vs. Foam | 3.375 | -30.70 to 37.45 | No | ns | 0.9986 |
| Coated Comp vs. TCPS | 72.30 | 38.23 to 106.4 | Yes | **** | <0.0001 |
| Foam vs. TCPS | 68.93 | 34.86 to 103.0 | Yes | **** | <0.0001 |

*Note: 6 well = 3.5 cm well; 12 well = 2.2-cm well; 24 well = 1.6-cm well.

**Table S4.** Two-way ANOVA probability values for Figure 5D.

| Tukey's multiple comparisons test | Mean Diff. | 95.00% CI of diff. | Below threshold? | Summary | Adjusted P Value |
|---|---|---|---|---|---|
| 0 | | | | | |
| Foam vs Coated | 0.000 | | | | |
| Foam vs Infused | 0.000 | | | | |
| Coated vs Infused | 0.000 | | | | |
| 15 | | | | | |
| Foam vs Coated | -0.1715 | -0.7189 to 0.3759 | No | ns | 0.5365 |
| Foam vs Infused | 0.05283 | -0.2044 to 0.3101 | No | ns | 0.7922 |
| Coated vs Infused | 0.2243 | -0.5080 to 0.9567 | No | ns | 0.3604 |
| 30 | | | | | |
| Foam vs Coated | -0.04717 | -0.6217 to 0.5274 | No | ns | 0.9680 |
| Foam vs Infused | 0.2605 | -0.2426 to 0.7636 | No | ns | 0.3023 |
| Coated vs Infused | 0.3077 | -0.3463 to 0.9616 | No | ns | 0.2022 |
| 60 | | | | | |
| Foam vs Coated | -0.1932 | -0.5520 to 0.1657 | No | ns | 0.3102 |
| Foam vs Infused | 0.08550 | -0.2624 to 0.4334 | No | ns | 0.7270 |
| Coated vs Infused | 0.2787 | -0.009487 to 0.5668 | No | ns | 0.0538 |
| 90 | | | | | |
| Foam vs Coated | -0.1160 | -0.4799 to 0.2479 | No | ns | 0.6186 |
| Foam vs Infused | 0.1253 | -0.4007 to 0.6513 | No | ns | 0.7414 |
| Coated vs Infused | 0.2413 | -0.3816 to 0.8643 | No | ns | 0.3114 |
| 120 | | | | | |
| Foam vs Coated | -0.08300 | -0.4055 to 0.2395 | No | ns | 0.7113 |
| Foam vs Infused | 0.1280 | -0.3774 to 0.6334 | No | ns | 0.7028 |
| Coated vs Infused | 0.2110 | -0.4451 to 0.8671 | No | ns | 0.3603 |
| 300 | | | | | |
| Foam vs Coated | -0.01250 | -0.09817 to 0.07317 | No | ns | 0.8937 |
| Foam vs Infused | 0.1345 | -0.3656 to 0.6346 | No | ns | 0.4786 |
| Coated vs Infused | 0.1470 | -0.4055 to 0.6995 | No | ns | 0.4301 |
| 600 | | | | | |
| Foam vs. Coated | -0.04100 | -0.2254 to 0.1434 | No | ns | 0.6672 |
| Foam vs. Infused | 0.1023 | -0.1918 to 0.3965 | No | ns | 0.3696 |
| Coated vs. Infused | 0.1433 | -0.1233 to 0.4099 | No | ns | 0.2320 |

**Table S5.** One-way ANOVA probability values for Figure 6C

| Tukey's multiple comparisons test | Mean Diff. | 95.00% CI of diff. | Below threshold? | Summary | Adjusted P Value |
|---|---|---|---|---|---|
| 0% vs. 20% | -9.104 | -10.87 to -7.340 | Yes | **** | <0.0001 |
| 0% vs. 40% | -9.128 | -10.89 to -7.365 | Yes | **** | <0.0001 |
| 0% vs. 60% | -10.17 | -11.93 to -8.403 | Yes | **** | <0.0001 |
| 0% vs. 100% | -4.987 | -6.751 to -3.223 | Yes | **** | <0.0001 |
| 0% vs. 80% | -14.52 | -16.29 to -12.76 | Yes | **** | <0.0001 |
| 20% vs. 40% | -0.02450 | -1.788 to 1.739 | No | ns | >0.9999 |
| 20% vs. 60% | -1.063 | -2.827 to 0.7012 | No | ns | 0.4475 |
| 20% vs. 100% | 4.117 | 2.353 to 5.881 | Yes | **** | <0.0001 |
| 20% vs. 80% | -5.421 | -7.185 to -3.657 | Yes | **** | <0.0001 |
| 40% vs. 60% | -1.038 | -2.802 to 0.7257 | No | ns | 0.4726 |
| 40% vs. 100% | 4.142 | 2.378 to 5.905 | Yes | **** | <0.0001 |
| 40% vs. 80% | -5.396 | -7.160 to -3.632 | Yes | **** | <0.0001 |
| 60% vs. 100% | 5.180 | 3.416 to 6.944 | Yes | **** | <0.0001 |
| 60% vs. 80% | -4.358 | -6.122 to -2.594 | Yes | **** | <0.0001 |
| 100% vs. 80% | -9.538 | -11.30 to -7.774 | Yes | **** | <0.0001 |

**Table S6.** One-way ANOVA probability values for Figure 6D

| Tukey's multiple comparisons test | Mean Diff. | 95.00% CI of diff. | Below threshold? | Summary | Adjusted P Value |
|---|---|---|---|---|---|
| 0% vs. 20% | -10.79 | -14.26 to -7.320 | Yes | **** | <0.0001 |
| 0% vs. 40% | -11.41 | -14.88 to -7.940 | Yes | **** | <0.0001 |
| 0% vs. 60% | -12.46 | -15.93 to -8.989 | Yes | **** | <0.0001 |
| 0% vs. 100% | -7.874 | -11.34 to -4.406 | Yes | **** | <0.0001 |
| 0% vs. 80% | -23.15 | -26.62 to -19.68 | Yes | **** | <0.0001 |
| 20% vs. 40% | -0.6206 | -4.089 to 2.847 | No | ns | 0.9931 |
| 20% vs. 60% | -1.669 | -5.137 to 1.799 | No | ns | 0.6746 |
| 20% vs. 100% | 2.914 | -0.5542 to 6.382 | No | ns | 0.1364 |
| 20% vs. 80% | -12.36 | -15.83 to -8.892 | Yes | **** | <0.0001 |
| 40% vs. 60% | -1.049 | -4.517 to 2.419 | No | ns | 0.9333 |
| 40% vs. 100% | 3.534 | 0.06638 to 7.003 | Yes | * | 0.0440 |
| 40% vs. 80% | -11.74 | -15.21 to -8.272 | Yes | **** | <0.0001 |
| 60% vs. 100% | 4.583 | 1.115 to 8.051 | Yes | ** | 0.0050 |
| 60% vs. 80% | -10.69 | -14.16 to -7.223 | Yes | **** | <0.0001 |
| 100% vs. 80% | -15.27 | -18.74 to -11.81 | Yes | **** | <0.0001 |

**Table S7.** Two-way ANOVA probability values for Figure 6E

| Šídák's multiple comparisons test | Mean Diff. | 95.00% CI of diff. | Below threshold? | Summary | Adjusted P Value |
|---|---|---|---|---|---|
| Before - After | | | | | |
| Thickness | -0.07300 | -0.4729 to 0.3269 | No | ns | 0.9588 |
| Length | 0.09200 | -0.3079 to 0.4919 | No | ns | 0.9222 |
| Width | 0.06900 | -0.3309 to 0.4689 | No | ns | 0.9649 |
